# Polymer-Assisted Crystallization and Defect Passivation in Planar Wide-Bandgap FAPbBr_3_ Perovskite Solar Cells

**DOI:** 10.1021/acsomega.5c04987

**Published:** 2025-09-04

**Authors:** Amalraj Peter Amalathas, Loheeswaran Selvadurai, Lucie Landová, Neda Neykova, Jakub Holovský

**Affiliations:** † Department of Physics, Faculty of Science, 70709 University of Jaffna, Jaffna 40000, Sri Lanka; ‡ Centre for Advanced Photovoltaics, Faculty of Electrical Engineering, 48220 Czech Technical University in Prague, Technická 2, 166 27 Prague, Czech Republic; § Department of Physical Science, Trincomalee Campus, Eastern University, Trincomalee 31010, Sri Lanka; ∥ Institute of Physics, Czech Academy of Sciences, v. v. i., Cukrovarnická 10, 162 00 Prague, Czech Republic

## Abstract

Wide-bandgap lead bromide perovskites such as FAPbBr_3_ are promising candidates for tandem solar cells and high-voltage optoelectronic applications, yet their performance is limited by surface and bulk defects that induce severe nonradiative recombination and limit stability. In this work, we present a defect passivation and crystallization control strategy by incorporating poly­(methyl methacrylate) (PMMA) into the antisolvent during FAPbBr_3_ film fabrication. PMMA treatment leads to improved film morphology with larger grains, reduced surface roughness, and enhanced crystallinity. FTIR analysis reveals that the carbonyl groups in PMMA coordinate with undercoordinated Pb^2+^ ions, effectively passivating electronic trap states. Photothermal deflection spectroscopy (PDS) shows reduced sub-bandgap absorption and lower Urbach energy, indicating suppressed deep-level defects and reduced energetic disorder. Enhanced photoluminescence intensity, prolonged carrier lifetimes, and decreased trap densities further confirm suppressed nonradiative recombination. As a result, PMMA treatment increases Voc by over 100 mV and improves power conversion efficiency by more than 1%, achieving a Voc of up to 1.510 V with reduced hysteresis and improved ambient stability. These findings demonstrate the effectiveness of polymer-assisted strategies for improving both efficiency and stability of wide-bandgap perovskite solar cells, offering a pathway toward high-voltage and tandem photovoltaic applications.

## Graphical abstract

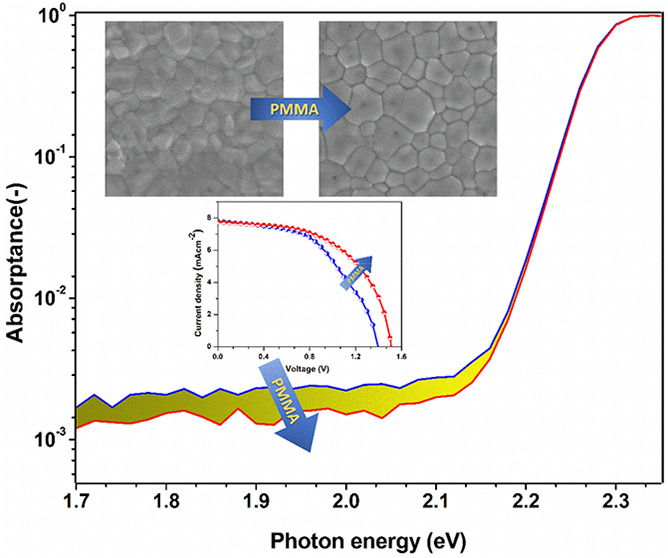

## 1 Introduction

Metal halide perovskites have revolutionized the field of photovoltaics and optoelectronics due to their exceptional optoelectronic properties, solution processability, and tunable bandgaps.^1–4^ Among these, wide-bandgap materials are especially promising for next-generation solar energy conversion, notably as top cells in tandem architectures designed to surpass the Shockley–Queisser limit of single-junction devices.^5–7^ Their bandgap tunability and high photovoltage potential also make them attractive for high-voltage optoelectronic applications such as photodetectors and LEDs.^8–11^ Formamidinium lead bromide (FAPbBr_3_), with a bandgap of approximately 2.28 eV, has drawn considerable attention for such applications due to its favorable optoelectronic properties and enhanced thermal and environmental stability compared to iodide-based counterparts.^12^

Despite these advantages, the power conversion efficiency (PCE) of FAPbBr_3_ based perovskite solar cells remains limited.^13^ One of the key challenges limiting the performance of FAPbBr_3_ PSCs is the formation of suboptimal perovskite films with poor crystallinity, small grain size, and inhomogeneous morphology.^14^ Achieving high quality perovskite thin films is intrinsically linked to the precise control of the perovskite crystallization process.^15^ Uncontrolled crystallization often results in films with poor morphology and a high density of defects. These morphological imperfections are closely associated with the generation of both bulk and surface defects.^14^ These defects act as nonradiative recombination centers, where photogenerated charge carriers are lost without contributing to the photocurrent or photovoltage. This nonradiative recombination leads to substantial losses in the open-circuit voltage (Voc), a critical parameter determining the overall power conversion efficiency of the solar cell.^16,17^ Furthermore, these defects can also compromise the long-term stability of the devices by providing pathways for material degradation.^18^ Therefore, mitigating these defects is essential for realizing the full potential of FAPbBr_3_ in high-performance solar cells.^19^

To address the detrimental effects of these defects in various perovskite materials, including FAPbBr_3_, several strategies have been investigated, such as compositional engineering, interface modification and the introduction of passivating agents.^19–24^ Among these strategies, the application of polymers as additives during perovskite film formation or as post-treatment layers has emerged as a promising strategy for defect passivation and crystallization control.^25–27^ Polymers, with their diverse chemical functionalities and ability to form thin films, can interact with the perovskite surface and grain boundaries, effectively passivating dangling bonds and ionic vacancies. Moreover, the presence of polymers during the film formation process can influence the nucleation and growth kinetics of the perovskite crystals, leading to improved film morphology with larger grains and reduced grain boundary density, which can further minimize defect-related recombination.^28^ While the use of poly­(methyl methacrylate) (PMMA) control has been explored in a range of perovskite materials, including those based on mixed halides and iodides^28–31^ for defect passivation and crystallization control, the specific incorporation of PMMA into the antisolvent during the fabrication of wide-bandgap FAPbBr_3_ perovskite solar cells to simultaneously achieve these benefits remains largely unexplored. Furthermore, most prior studies have assessed the benefits of PMMA indirectly through device metrics or photoluminescence, with little to no direct evidence of deep-level defect passivation, particularly in FAPbBr_3_.

In this study, we address this critical gap by providing, for the first time, direct spectroscopic evidence of deep defect passivation in FAPbBr_3_ perovskite films treated with PMMA via the antisolvent route. Specifically, we employ Photothermal Deflection Spectroscopy (PDS) to probe sub-bandgap absorption, revealing a substantial suppression of deep-level states upon PMMA treatment. We further quantify the reduction in energetic disorder via a measurable decrease in Urbach energy, demonstrating a transition toward a more electronically ordered material. We further explore the interaction mechanism between PMMA and FAPbBr_3_, alongside detailed analyses of film morphology, crystallinity, defect density, and charge carrier dynamics. The results demonstrate a significant reduction in nonradiative recombination, leading to a substantial increase in Voc and efficiency with negligible hysteresis, highlighting the potential of this approach for high-performance wide-bandgap FAPbBr_3_ perovskite solar cells.

## 2 Results and Discussion

The surface morphology of FAPbBr_3_ perovskite films prepared without and with PMMA antisolvent treatment was examined by scanning electron microscopy (SEM) and atomic force microscopy (AFM), as shown in Figure 1. Low-magnification SEM images (Figure 1a,b) indicate that both films exhibit continuous and uniform coverage; however, clear differences emerge at higher magnifications (Figure 1c,d). Specifically, the control film (without PMMA, Figure 1c) shows relatively small grains and distinct grain boundaries. In contrast, the PMMA-treated film (Figure 1d) exhibits significantly larger, densely packed grains, resulting in fewer grain boundaries and improved morphological uniformity. AFM topographical analysis (Figure 1e,f) further confirms the improved surface characteristics induced by PMMA treatment. The calculated root-mean-square (RMS) surface roughness decreases from 16.8 nm in the untreated sample to 12.3 nm in the PMMA-treated film, indicating a smoother surface topography. This reduced roughness is beneficial for charge extraction and transport at device interfaces, suggesting that PMMA incorporation during the antisolvent step effectively enhances film quality. Overall, these results clearly demonstrate that introducing PMMA into the antisolvent step significantly improves the crystallization process, promoting the growth of larger grains and producing smoother and more uniform perovskite films, which are essential for minimizing defect states and enhancing device performance.^32^

**Figure 1:**
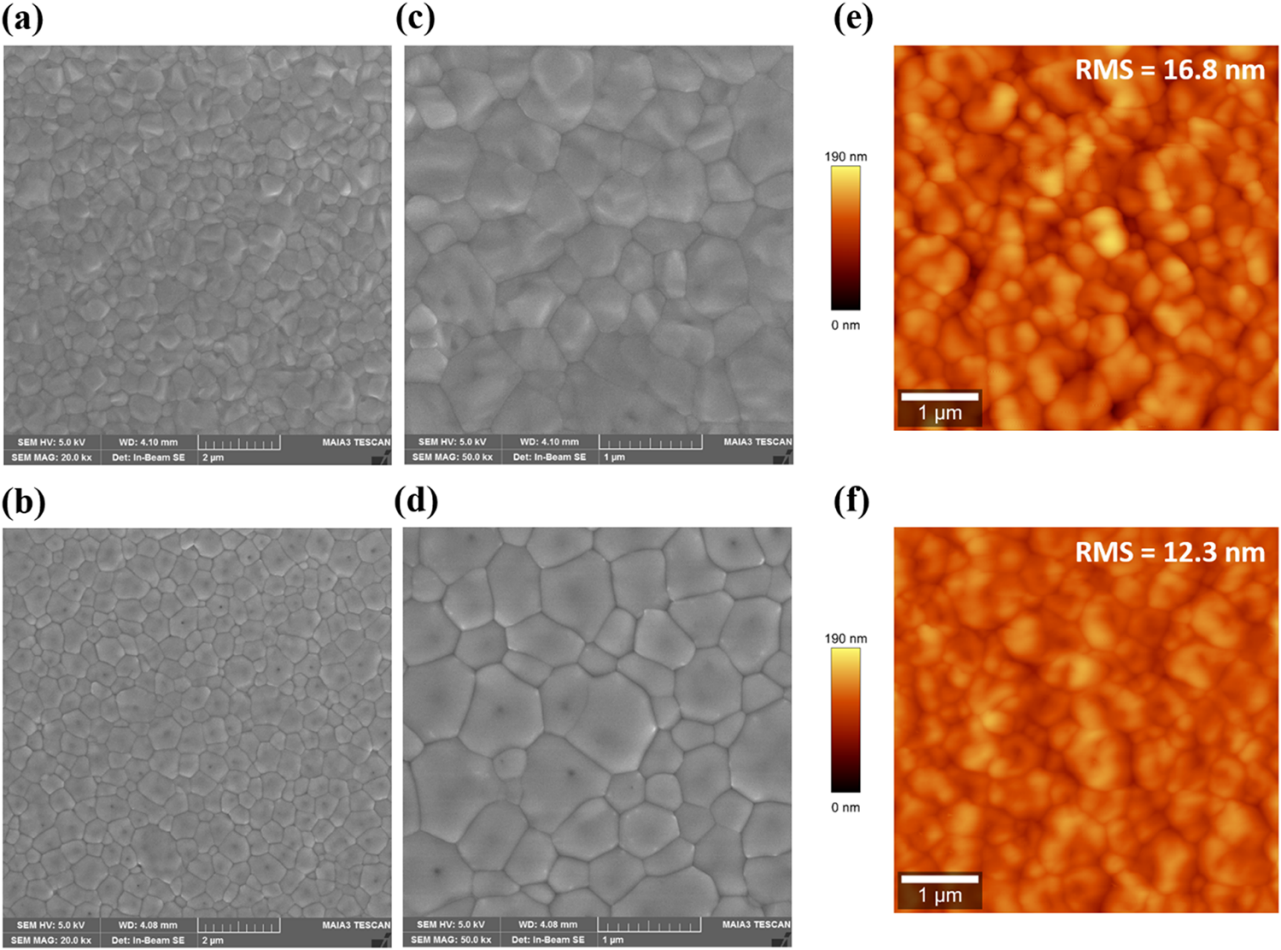
Surface morphology of FAPbBr_3_ perovskite films fabricated without (top row) and with (bottom row) PMMA antisolvent treatment. (a, b) Low-magnification SEM images, (c, d) high-magnification SEM images showing differences in grain size and uniformity, and (e, f) AFM images of the corresponding films.

To evaluate the optical absorption behavior and determine the optical bandgap of the perovskite films, UV–vis absorption spectra were recorded and analyzed using Tauc plots. As shown in the Figure S1, both untreated and PMMA-treated FAPbBr_3_ films exhibit similar absorption profiles with a steep onset near 540 nm. The optical bandgaps for both films are approximately 2.28 eV, consistent with literature values for FAPbBr_3_, indicating that PMMA incorporation does not alter the electronic band structure.^33^

X-ray diffraction (XRD) measurements were conducted to analyze the crystalline phase and crystallinity of the FAPbBr_3_ films prepared without and with PMMA antisolvent treatment, as shown in Figure 2. Both films show clear diffraction peaks at approximately 14.8°, 20.9°, 29.8°, and 33.4°, corresponding to the (001), (011), (002), and (012) crystal planes, respectively, indicating the formation of a well-defined crystalline perovskite structure. Notably, the PMMA-treated film demonstrates stronger diffraction intensity, especially for the dominant (001) peak, suggesting an improvement in crystallinity due to PMMA incorporation. Further examination of the prominent (001) diffraction peaks (Figure 2b) reveals that the full width at half-maximum (FWHM) decreases from 0.0649° for the untreated film to 0.0588° for the PMMA-treated film. Based on Scherrer’s equation, this reduction in peak width corresponds to an increase in crystallite size from approximately 128.9 to 142.2 nm, indicating enhanced crystallinity and improved crystalline quality upon PMMA treatment. The larger crystallite size observed with PMMA treatment aligns well with the SEM observations^34^ of larger grains and reduced grain boundaries, supporting the conclusion that PMMA effectively regulates the crystallization kinetics, facilitating more controlled nucleation and grain growth processes. It is worth noting that the (001) diffraction peak of the pristine FAPbBr_3_ film exhibits slight asymmetry. This asymmetry may arise from microstrain within the crystal lattice, variation in grain size, or a minor distribution in crystallite orientation, indicating a certain degree of structural heterogeneity in the untreated film. Such irregularities could potentially lead to increased trap densities and nonradiative recombination pathways, thereby affecting charge transport properties. In contrast, the sharper and more symmetric (001) peak observed in the PMMA-treated film suggests reduced structural disorder and improved phase purity. These structural improvements are expected to positively influence device performance by enhancing charge carrier mobility and reducing recombination losses, ultimately contributing to higher efficiency and stability.^34^

**Figure 2:**
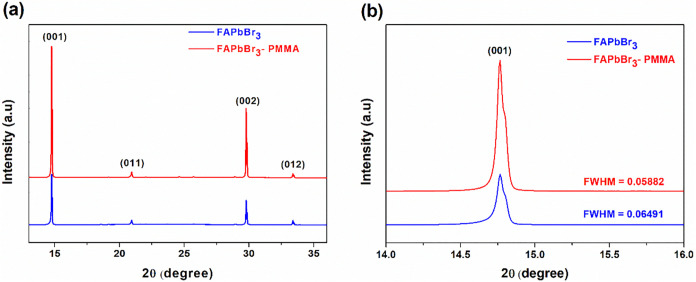
X-ray diffraction (XRD) patterns of FAPbBr_3_ perovskite films fabricated without and with PMMA antisolvent treatment. (a) Full XRD spectra showing diffraction peaks corresponding to characteristic crystal planes. (b) Magnified view of the prominent (001) diffraction peaks, highlighting changes in full width at half-maximum (FWHM).

FTIR spectroscopy was performed to investigate the chemical interactions PMMA and FAPbBr_3_ perovskite precursors, specifically focusing on how PMMA coordinates with lead bromide (PbBr_2_) and formamidinium bromide (FABr). Figure 3a shows the FTIR spectra of pristine PMMA, and PMMA mixed separately with PbBr_2_ and FABr. Pure PMMA exhibits a distinct and intense absorption band at approximately 1730 cm^–1^, characteristic of the carbonyl (C=O) stretching vibration. Upon mixing PMMA with PbBr_2_, the carbonyl absorption peak shifts significantly to a lower wavenumber (1719 cm^–1^), indicating a notable weakening of the C=O bond. This shift results from the coordination interaction between the electron-rich carbonyl group and electron-deficient Pb^2+^ ions, demonstrating a Lewis acid–base interaction.^28^ Such interactions are essential as they effectively passivate undercoordinated Pb^2+^ ions, a known source of deep-level electronic trap states that severely impact perovskite device performance.^35^ Similarly, when PMMA is mixed with FABr, the carbonyl peak shifts even further, down to 1716 cm^–1^, confirming an additional or stronger interaction with the organic cation (FA^+^). This indicates that the carbonyl group in PMMA interacts not only with the inorganic precursor (Pb^2+^ ions) but potentially also through hydrogen bonding or dipolar interactions with the organic FA^+^ species.^30^ Such multiple-site coordination effectively immobilizes precursor species, thus promoting controlled crystallization and better film formation. These shifts in FTIR spectra conclusively indicate that the carbonyl groups in PMMA engage strongly with the perovskite precursors through Lewis acid–base and/or polar interactions, thereby chemically passivating defects at grain boundaries and interfaces.^19^ This chemical passivation effectively reduces the density of trap states, mitigating nonradiative recombination pathways and contributing to improved device characteristics such as higher open-circuit voltage and enhanced stability.^36^

**Figure 3:**
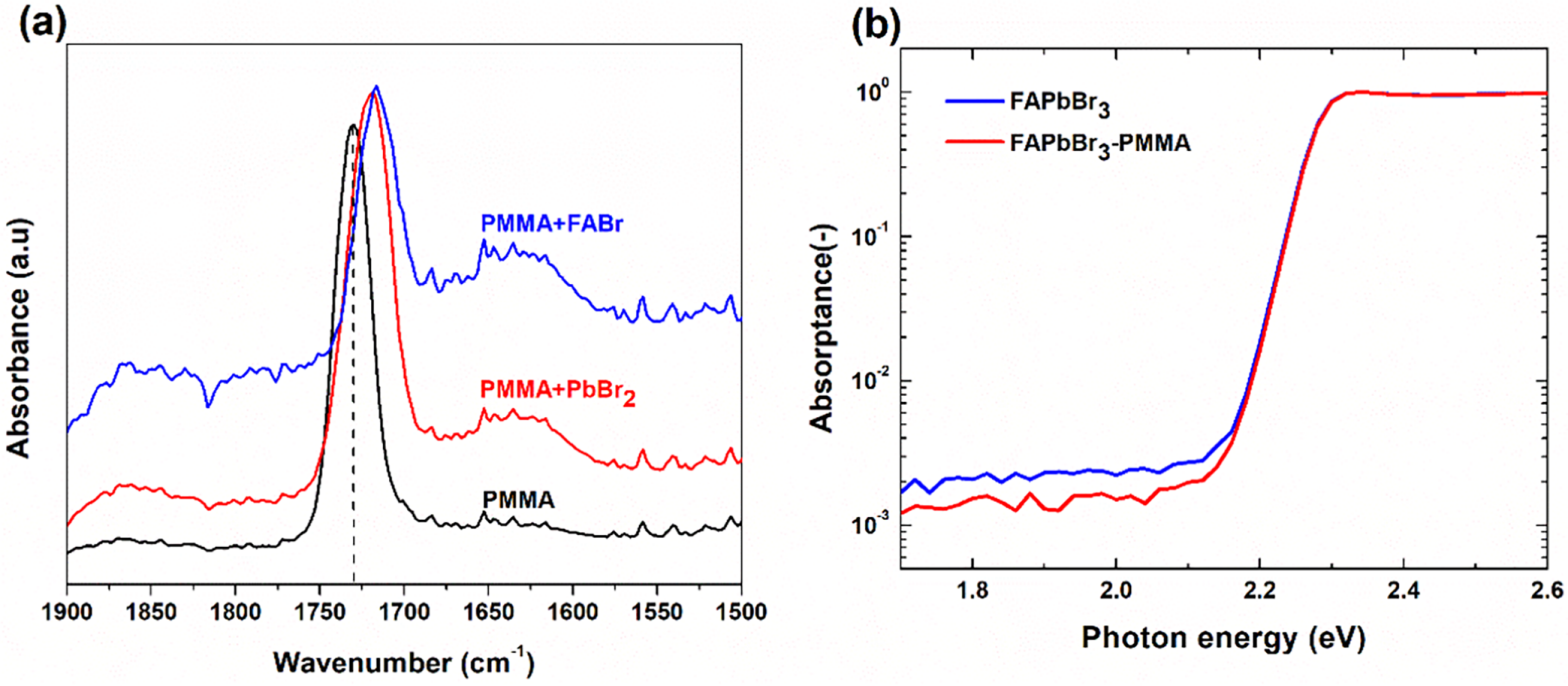
(a) FTIR spectra of pristine PMMA, PMMA mixed with PbBr_2_, and PMMA mixed with FABr, highlighting shifts in the carbonyl stretching vibration peak and (b) photothermal deflection spectroscopy (PDS) spectra showing sub-bandgap absorptance of FAPbBr_3_ films fabricated without and with PMMA antisolvent treatment.

Photothermal deflection spectroscopy (PDS) measurements were performed to investigate the influence of PMMA on deep defect states and energetic disorder in the FAPbBr_3_ perovskite films. Figure 3b compares the sub-bandgap absorptance spectra of untreated and PMMA-treated FAPbBr_3_ perovskite films. Both films exhibit an exponential absorption tail below the bandgap, indicative of the presence of electronic states extending into the forbidden gap, commonly originating from defects such as undercoordinated ions and vacancies. The PMMA-treated films show significantly reduced sub-bandgap absorptance compared to the untreated control. This suppressed sub-bandgap absorption suggests that PMMA passivation effectively reduces the density of deep-level defects, consistent with the observed chemical interactions confirmed via FTIR analysis. To further quantify the impact of PMMA on energetic disorder within the films, the Urbach energy (Eu) was extracted from the exponential region of the absorption tail (Figure S2).^37,38^ The untreated FAPbBr_3_ film shows an Eu of 25.3 meV, reflecting a comparatively higher degree of energetic disorder. In contrast, the PMMA-treated film exhibited a lower Eu of 24.5 meV, clearly indicating a reduction in disorder and thus improved electronic structure. The decrease in Eu aligns closely with improvements observed in morphology and crystallinity from SEM, AFM, and XRD analyses, highlighting that PMMA incorporation during film formation yields more uniform crystal growth, larger grains, and reduced grain boundary regions. Such morphological and structural improvements effectively limit the formation of defect states and consequently reduce energetic disorder, facilitating more efficient charge transport and lower recombination rates.

To further elucidate the influence of PMMA incorporation on the charge carrier dynamics in FAPbBr_3_ perovskite films, steady-state photoluminescence (PL) and time-resolved photoluminescence (TRPL) measurements were performed (Figure 4). The PL spectra as shown in Figure 4a reveal a significantly enhanced emission intensity for PMMA-treated films compared to the untreated films. This notable increase in PL intensity indicates reduced nonradiative recombination rates, consistent with effective passivation of defect sites by PMMA, as supported by previous FTIR and PDS analyses. TRPL measurements were performed to gain deeper insight into carrier recombination dynamics. The TRPL decay curves as shown in Figure 4b demonstrate prolonged carrier lifetime for PMMA-treated films compared to the control. The detailed fitted parameters and average PL lifetime are summarized in Table S1. The average carrier lifetime increases from approximately 27 ns in the untreated film to 36 ns in the PMMA-treated film. This substantial enhancement in carrier lifetime strongly suggests reduced nonradiative recombination, which can be attributed to effective passivation of both surface and bulk defect states by PMMA. The extended carrier lifetime facilitates more efficient charge extraction, which is crucial for improving photovoltaic device performance, particularly open-circuit voltage and efficiency.^34^

**Figure 4:**
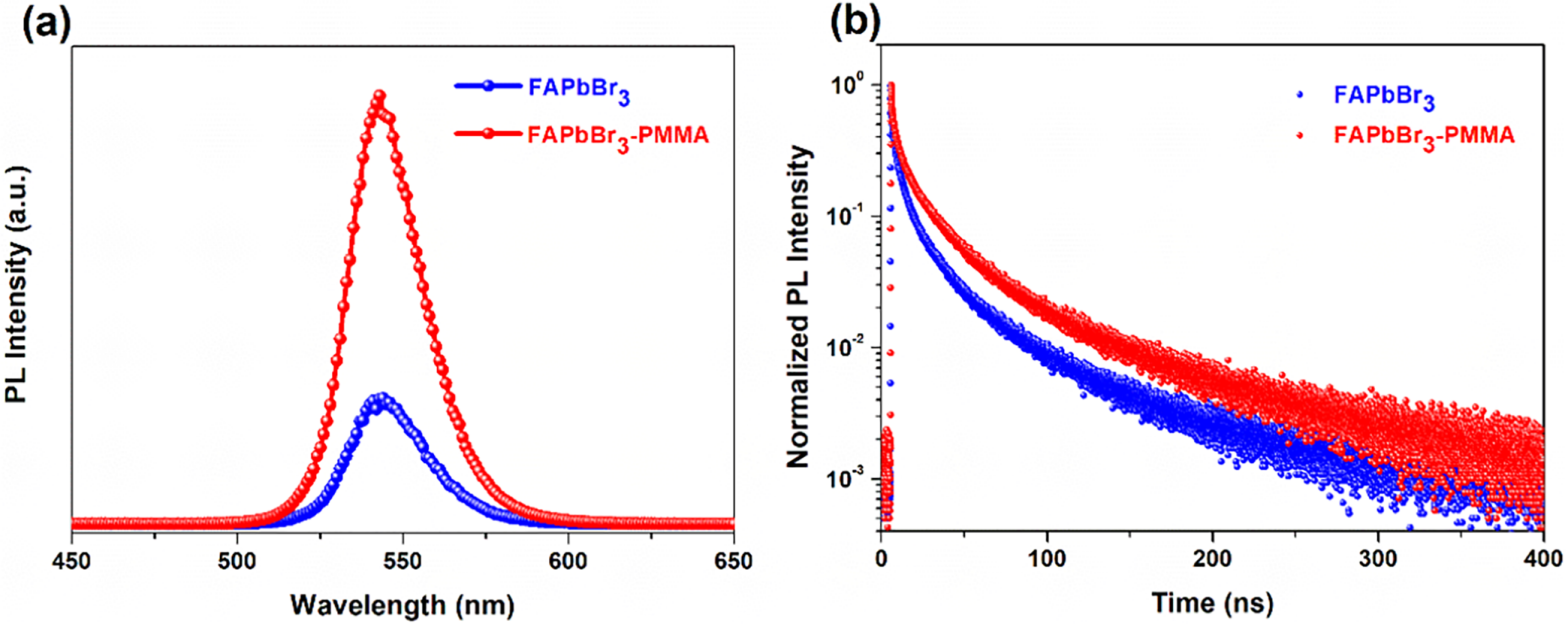
(a) Steady-state PL spectra and (b) TRPL decay curves of FAPbBr_3_ films prepared without and with PMMA antisolvent treatment.

To quantitatively evaluate the defect density reduction, space-charge-limited current (SCLC) measurements were performed (Figure S3).^39,40^ For the untreated FAPbBr_3_ films, the trap-filled limit voltage (*V*_TFL_) of 0.42 V was observed, corresponding to a defect density (*N_t_*) of approximately 1.09 × 10^16^ cm^–3^. In contrast, PMMA-treated films exhibit a significantly lower *V*_TFL_ of 0.25 V, reflecting a reduced defect density of about 5.00 × 10^15^ cm^–3^, approximately an order of magnitude lower than that of the untreated sample. This substantial reduction in defect density aligns with the enhanced PL intensity and longer carrier lifetimes, reinforcing the effectiveness of PMMA passivation. Collectively, the PL, TRPL, and SCLC results clearly demonstrate that PMMA treatment significantly suppresses nonradiative recombination by effectively reducing the density of electronic trap states within the FAPbBr_3_ films. These results confirm the dual beneficial role of PMMA in both crystallization control and defect passivation, ultimately contributing to the improved photovoltaic performance of planar wide-bandgap perovskite solar cells.

The photovoltaic performance of planar FAPbBr_3_ solar cells with and without PMMA antisolvent treatment was evaluated through current density–voltage (*J*–*V*) measurements under AM 1.5G illumination. Figure 5a shows the *J*–*V* curves of the best-performing devices from each set. The PMMA-treated device achieves a significantly improved Voc of 1.510 V, compared to 1.395 V in the untreated device. This substantial enhancement in Voc by more than 100 mV clearly reflects the reduced nonradiative recombination and defect states as previously observed from PL, TRPL, and SCLC analyses. Additionally, the PMMA-treated cell exhibits improvements in fill factor (FF) from 51.3% to 55.4%, contributing to an overall increase in PCE from 5.57% to 6.48%. Integrated Jsc values from EQE curves closely match those obtained from *J*–*V* measurements, validating the accuracy and consistency of the reported device performances (Figure S4). The statistical analysis of photovoltaic parameters from multiple devices (Figures 5b, S5, and Table S2) confirms the consistent improvement in device performance upon PMMA treatment. PMMA-treated devices demonstrate notably enhanced and more reproducible Voc, FF, and overall PCE compared to untreated devices, highlighting the effectiveness of PMMA in reducing defects and improving device uniformity. The hysteresis behavior of the best-performing devices was investigated by comparing forward and reverse *J*–*V* scans (Figure S6, and Table S3). The untreated device shows significant hysteresis with a hysteresis index (H-index) of 13.8%, while the PMMA-treated device exhibits substantially reduced hysteresis (5.1%), confirming the effectiveness of defect passivation and enhanced charge extraction. This reduced hysteresis further emphasizes improved interfacial quality and minimized ion migration within the PMMA-treated perovskite films.

**Figure 5:**
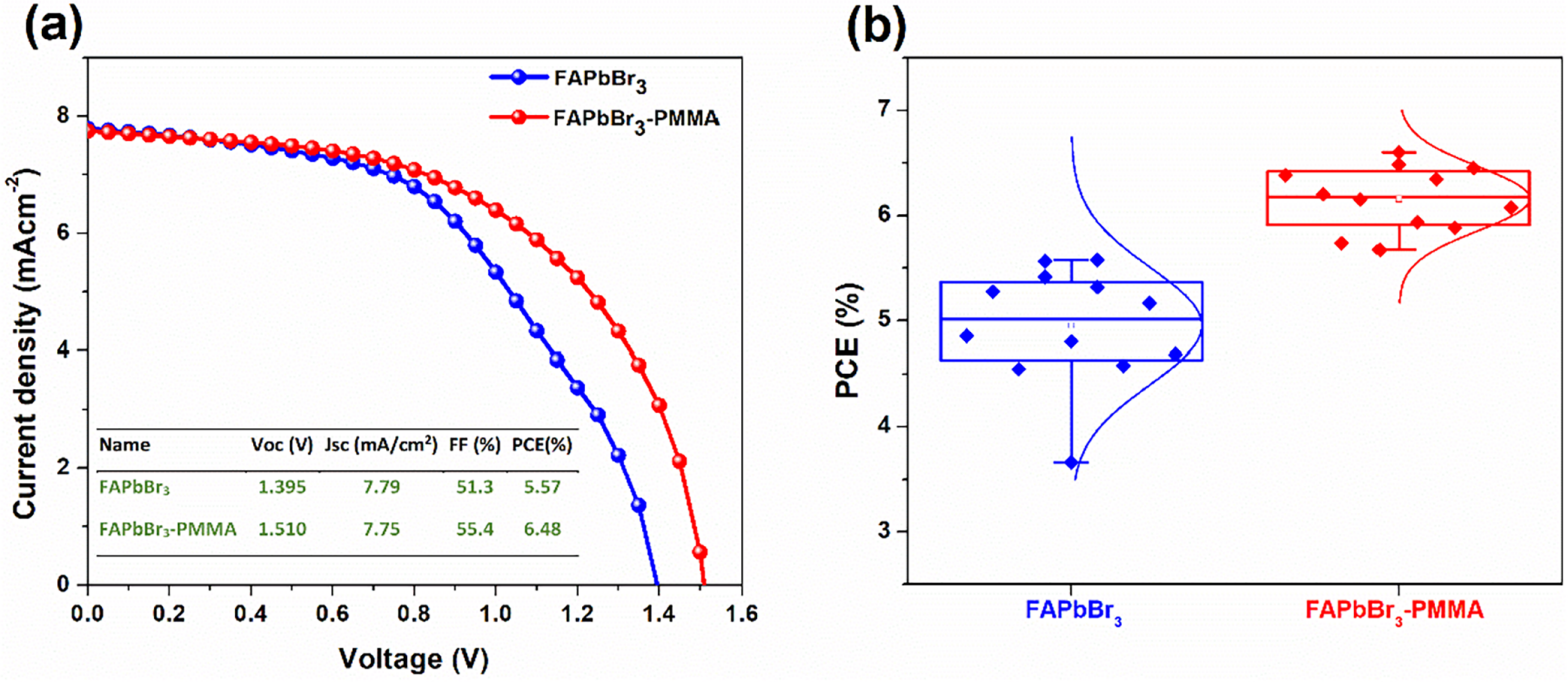
(a) *J*–*V* curves of the best-performing FAPbBr_3_ solar cells without and with PMMA antisolvent treatment and (b) box plots showing the statistical distribution of PCE values from multiple devices.

To assess the environmental stability of the perovskite solar cells, the best-performing devices were stored under ambient dark conditions for 4 weeks, and their photovoltaic performance was remeasured. The *J*–*V* characteristics of both untreated and PMMA-treated FAPbBr_3_ devices before and after aging are shown in Figure 6. The untreated FAPbBr_3_ device shows noticeable degradation, with its PCE dropping from 5.57% to 4.34%, corresponding to a PCE retention of only 77.9% (Table S4). In contrast, the PMMA-treated device maintained significantly better stability, retaining 91.2% of its initial PCE (from 6.48% to 5.91%) after 4 weeks. This improved stability is attributed to the passivating and encapsulating effects of PMMA. Its carbonyl groups coordinate with undercoordinated Pb^2+^ ions, suppressing defect-assisted degradation pathways. Additionally, PMMA likely serves as a moisture barrier, minimizing environmental-induced degradation.

**Figure 6:**
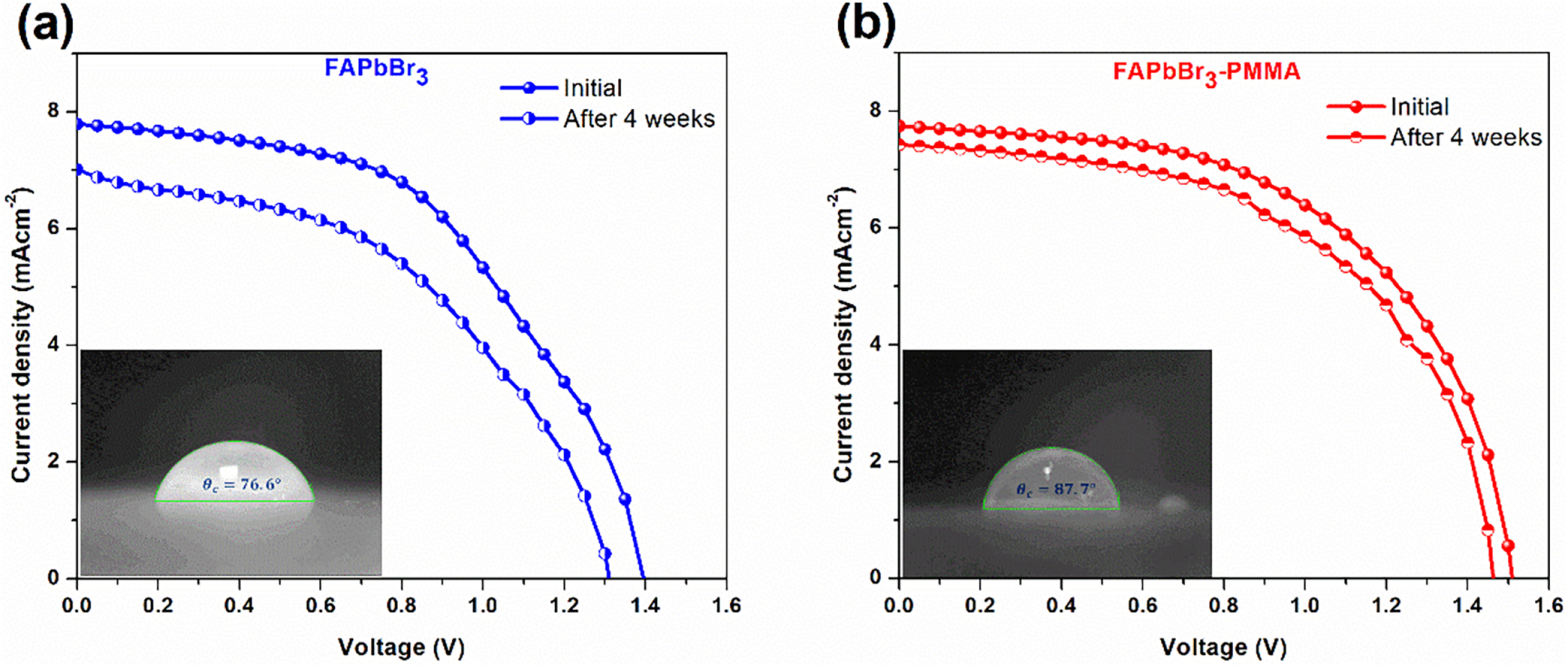
*J*–*V* curves of the best-performing FAPbBr_3_ devices (a) without and (b) with PMMA antisolvent treatment, measured initially and after 4 weeks of storage under ambient dark conditions. Insets show the corresponding water contact angles on perovskite films.

To further probe the surface protection effect, water contact angle measurements were performed on the perovskite films (insets of Figure 6a,b). The untreated film exhibits a contact angle of 76.6°, while the PMMA-treated film shows a significantly higher angle of 87.7°, indicating enhanced surface hydrophobicity. This increased water repellency further supports the role of PMMA in improving the ambient stability of FAPbBr_3_ films by limiting moisture ingress. Together, these results demonstrate that incorporating PMMA into the antisolvent not only improves initial device performance but also provides enhanced environmental stability by reducing defect density and increasing surface hydrophobicity. Overall, the systematic photovoltaic characterization clearly highlights that the incorporation of PMMA into the antisolvent step enhances device efficiency, stability, reproducibility, and reduces hysteresis, validating its dual role in controlling crystallization and passivating defects in planar FAPbBr_3_ perovskite solar cells.

To better understand the significance of our results and to place our work in the context of recent developments, we have compared the key performance metrics of our devices with those of other reported high performing FAPbBr_3_ based solar cells. While a comprehensive summary of both single step and two step fabrication methods is provided in the Supporting Information (Table S5), particular attention is given to single step methods, which are most directly comparable to our approach. Recent studies have achieved impressive PCEs exceeding 10% using advanced approaches such as additive engineering or tailored solvent systems. For instance, Yue et al. employed an intermediate-phase transition-assisted blade coating technique to reach a PCE of 10.86%,^41^ while Zhu et al. achieved 11.4% using specially designed self-assembled monolayers (SAMs).^42^ However, these high efficiencies often come at the cost of more complex fabrication procedures or customized material synthesis. In contrast, one of the key advantages of our approach is the high Voc of 1.510 V, which is among the highest reported for devices based on single step methods. This improvement is attributed to reduced nonradiative recombination, as supported by our PDS and TRPL results. Although some reports using additives like guanidinium bromide have achieved even higher Voc values close to 1.64 V, our method offers a significant voltage enhancement using a much simpler strategy. Most importantly, our approach stands out for its simplicity. By simply adding a widely available polymer (PMMA) to the antisolvent, we achieve balanced improvements in all key device parameters without the need for novel passivation molecules or complex multilayer structures. Our device not only delivers a high Voc and a solid PCE of 6.48%, but also shows excellent stability, retaining 91% of its original efficiency after 4 weeks under ambient conditions. These results demonstrate that a straightforward modification to a standard single step process can be an effective and scalable route for improving the performance of wide-bandgap perovskite solar cells.

## 3 Conclusions

In summary, we demonstrate that incorporating PMMA into the antisolvent is a simple yet highly effective strategy to simultaneously control crystallization and passivate defects in planar FAPbBr_3_ perovskite solar cells. This approach results in improved film morphology, enhanced crystallinity, reduced sub-bandgap defect states, and lower energetic disorder, as confirmed by comprehensive structural, optical, and electrical characterizations. Consequently, PMMA-treated devices exhibit significantly enhanced photovoltaic performance, including higher Voc, improved fill factor, and better efficiency, along with reduced hysteresis and noticeably improved ambient stability. These results highlight the potential of polymer-assisted defect management in advancing wide-bandgap perovskites for high-voltage and tandem solar cell applications.

## 4 Materials and Methods

### 4.1 Materials and Solvents

The ITO-coated glass substrates (sheet resistance 7–10 ohm/sq) were obtained from MSC Supplies. Tin­(IV) oxide (SnO_2_) colloidal dispersion in water (15 wt %) was purchased from Alfa Aesar. The hole transport material 2,2′,7,7′-tetrakis­(*N*,*N*-di-4-methoxyphenylamino)-9,9′-spirobifluorene (Spiro-MeOTAD) was purchased from Lumtec. Potassium chloride (KCl), lead­(II) bromide (PbBr_2_), formamidinium bromide­(FABr), methanol anhydrous, *N*,*N*-dimethylformamide (DMF), dimethyl sulfoxide (DMSO), poly­(methyl methacrylate) (PMMA), chlorobenzene (CB), lithium bis­(trifluoromethanesulfonyl)­imide (Li-TFSI), pyridine (t-BP) and acetonitrile (ACN) were purchased from Sigma-Aldrich. [6,6]-phenyl C61 butyric acid methyl ester (PCBM) was obtained from Special Carbon. All the materials and solvents were used as received without further purification.

### 4.2 Fabrication of FAPbBr_3_ Perovskite Solar Cells

ITO-coated glass substrates were cleaned by sonication in a 2% (v/v) Hellmanex detergent solution prepared in deionized (DI) water for 30 min. Following a thorough rinse with DI water, the substrates were sequentially sonicated in acetone and isopropanol (IPA) for 20 min each. The cleaned substrates were dried under a nitrogen stream and subsequently treated with UV-ozone for 15 min. To prepare the electron transport layer, a SnO_2_ precursor solution was made by diluting a 15 wt % aqueous colloidal dispersion of SnO_2_ with DI water in a 1:4 volume ratio. The 2.5 mg of KCl was dissolved in 1 mL of the diluted SnO_2_ solution, yielding a 2.5 mg/mL KCl mixed SnO_2_ solution. This mixed solution was spin-coated onto the precleaned ITO substrates at 4000 rpm for 30 s. The films were then annealed at 150 °C for 30 min in ambient air (relative humidity: 40–50%). After cooling to room temperature, the substrates underwent a second UV-ozone treatment for 15 min before being transferred into a nitrogen-filled glovebox for further processing. The FAPbBr_3_ perovskite precursor solution was prepared by dissolving 1.2 mmol of FABr and 1.2 mmol of PbBr_2_ in 1 mL of a mixed solvent system consisting of DMF and DMSO in a 4:1 volume ratio (v/v). The solution was stirred at 60 °C overnight and then filtered using a 0.2 μm PTFE syringe filter prior to use. The perovskite solution was spin-coated on SnO_2_ films at 1000 rpm for 10 s and 4000 rpm for 30 s. During the second spin step, 150 μL of CB was used as the antisolvent and dropped onto the substrate 10 s before the end of the spin process. The coated films were then annealed at 80 °C for 5 min, followed by 150 °C for 20 min. To fabricate FAPbBr_3_ perovskite films with PMMA incorporated via the antisolvent method, 1 mg of PMMA was dissolved in 1 mL of CB and this PMMA-containing CB solution was used as the antisolvent following the same deposition procedure described above. After the perovskite layer cooled to room temperature, the hole transport layer was deposited by spin-coating Spiro-MeOTAD solution at 4000 rpm for 30 s. The Spiro-MeOTAD solution was prepared by dissolving Spiro-MeOTAD (72.3 mg/mL) in CB, followed by the addition of 28.8 μL of t-BP and 17.5 μL of a Li-TFSI solution (520 mg Li-TFSI dissolved in 1 mL of ACN). The completed devices were stored overnight in a dry box before depositing an 80 nm Au electrode using thermal evaporation under high vacuum. A shadow mask was used to define the electrode pattern.

### 4.3 Electron-Only Device Fabrication for SCLC Measurements

Electron-only devices were fabricated with the architecture: ITO/SnO_2_/FAPbBr_3_ (with or without PMMA in the antisolvent)/PCBM/Au. For the electron transport layer, a PCBM solution (10 mg/mL in CB) was spin-coated onto the perovskite layer at 4000 rpm for 30 s and annealed at 100 °C for 10 min. The procedures for depositing SnO_2_ and FAPbBr_3_ (with and without PMMA in the antisolvent) were identical to those described above.

### 4.4 Materials and Device Characterization

The morphology of the perovskite films was examined using a field emission scanning electron microscope (Tescan MAIA) operated at an accelerating voltage of 5 kV. Atomic force microscopy (AFM) images were obtained using a WItec α 300ARS, a commercial system integrating AFM, scanning near-field optical microscopy, and Raman spectroscopy. The crystallinity of the perovskite films was analyzed via X-ray diffraction (XRD) using a PANalytical AERIS benchtop diffractometer with Cu Kα_1_ radiation (λ = 0.15406 nm). UV–vis absorption spectra were recorded with a Bentham system equipped with a TMC 300 monochromator and a 50 W tungsten halogen lamp. Photothermal deflection spectroscopy (PDS) was conducted using an in-house setup capable of simultaneously measuring transmittance and reflectance to probe sub-bandgap absorption features, with Fluorinert FC-72 serving as the thermal probe liquid. Fourier-transform infrared spectroscopy (FTIR) measurements were carried out using a Thermo Nicolet Nexus 870 FTIR spectrophotometer. Steady-state photoluminescence (PL) spectra were recorded with a Cary Eclipse fluorescence spectrophotometer under 300 nm excitation. Time-resolved PL decay measurements were performed using a FluoTime 200 system (PicoQuant GmbH), excited by a 442 nm picosecond pulsed laser. The current density–voltage (*J*–*V*) characteristics were measured under simulated AM 1.5G solar illumination (100 mW/cm^2^) using the LED solar simulator (Wavelabs Solar Metrology Systems) and a computer-controlled Keithley 2400 source meter. A metal mask defining an active area of 0.09 cm^2^ was used during *J*–*V* measurements. External quantum efficiency (EQE) measurements were carried out with a Bentham system consisting of a TMC 300 monochromator, 50 W tungsten halogen lamp, and a lock-in amplifier, using a calibrated silicon photodiode as the reference. The current–voltage characteristics of the electron-only devices were measured in the dark using the same Keithley 2400 source meter. All measurements were conducted at room temperature under ambient conditions without encapsulation.

## Supplementary Material



## Notes

Tauc plots; extraction of Urbach energy; calculated parameters of the TRPL spectra; SCLC measurements; EQE spectra and integrated *J*_SC_; box plots of PV parameters; average photovoltaic parameters; (*J*–*V*) curves and photovoltaic parameters under reverse and forward; photovoltaic parameters of initial and after 4 weeks; summary of the reported work (PDF)
