# Supplementary material for: Polymer-Assisted Crystallization and Defect Passivation in Planar Wide-Bandgap FAPbBr3 Perovskite Solar Cells
Source: ACS Omega. 2025 Sep 4;10(36):41515–23. doi: 10.1021/acsomega.5c04987 (PMC12444675; doi:10.1021/acsomega.5c04987)
Supplement: Supplementary file 1 [file ao5c04987_si_001.pdf]

Supporting Information:

Polymer-assisted Crystallization and Defect Passivation in Planar Wide Bandgap $FAPbBr_3$ Perovskite Solar Cells

Amalraj Peter Amalathas[1,2*], Loheeswaran Selvadurai[1,3], Lucie Landová[2,4], Neda Neykova[2,4] and Jakub Holovský[2,4*]

[1]*Department of Physics, Faculty of Science, University of Jaffna, Jaffna 40000, Sri Lanka*

[2]*Centre for Advanced Photovoltaics, Faculty of Electrical Engineering, Czech Technical University in Prague, Technická 2, 166 27 Prague, Czech Republic*

[3]*Department of Physical Science, Trincomalee Campus, Eastern University, Trincomalee 31010, Sri Lanka*

[4] *Institute of Physics, Czech Academy of Sciences, v. v. i., Cukrovarnická 10, 162 00 Prague, Czech Republic*

**Corresponding authors: amalraj@univ.jfn.ac.lk; holovsky@fzu.cz*

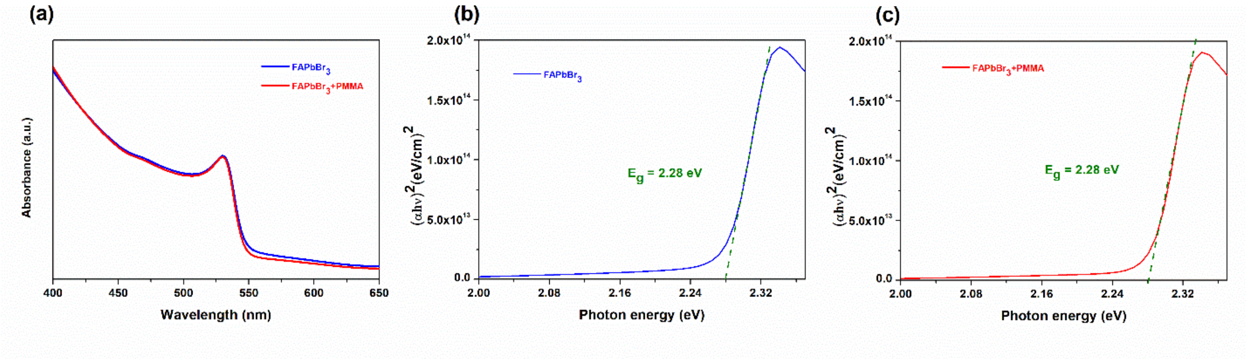

Figure S1: (a) UV–Vis absorbance spectra of $FAPbBr_3$ films with and without PMMA treatment and (b, c) Tauc plots used to estimate the optical bandgap (Eg), showing Eg ≈ 2.28 eV for both samples.

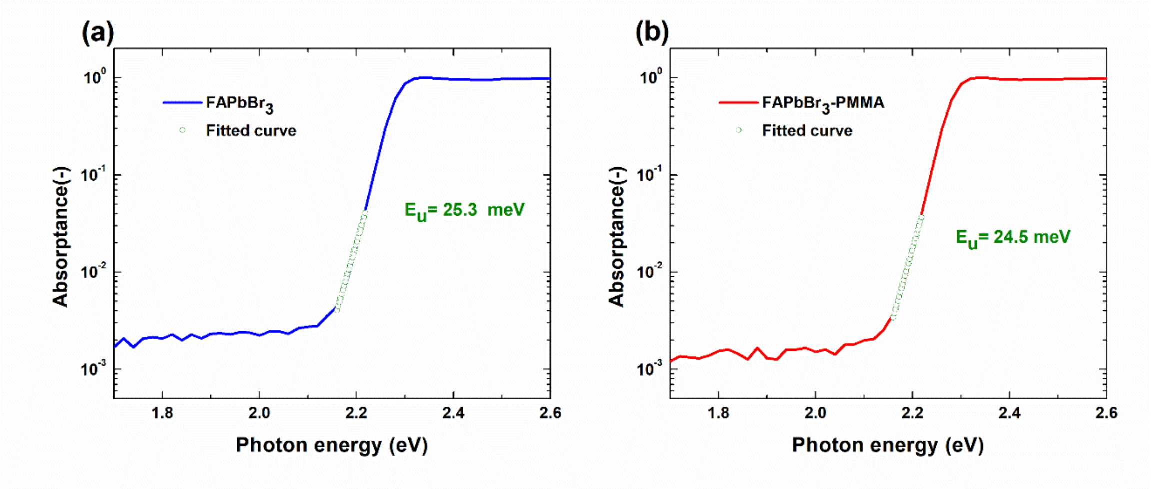

Figure S2: Extraction of Urbach energy (E_u) from PDS spectra for (a) untreated and (b) PMMA-treated $FAPbBr_3$ films, demonstrating reduced energetic disorder upon PMMA incorporation.

The carrier lifetime is obtained by fitting the PL transient decays with a bi-exponential decay function as follows

$$f(t) = A_1 exp^{(-\frac{t}{\tau_1})} + A_2 exp^{(-\frac{t}{\tau_2})} + B$$

where A_1 and A_2 represent the time-independent decay amplitudes, B is a constant, and τ_1 and τ_2 are the fast and slow decay time, respectively. The weighted-average lifetime (τ_{avg}) is calculated from the fit curve parameters according to the following equation.

$$\tau_{avg} = \frac{A_1 {\tau_1}^2 + A_2 {\tau_2}^2}{A_1 \tau_1 + A_2 \tau_2}$$

Table S1. Summary of the fit and calculated parameters of the TRPL spectra of the $FAPbBr_3$ perovskite films prepared without and with PMMA anti-solvent treatment.

Sample name	A_1(%)	τ_1 (ns)	A_2(%)	τ_2 (ns)	Average τ (ns)
$FAPbBr_3$	83.40	5.88	16.60	41.95	27.05
$FAPbBr_3$-PMMA	77.40	8.30	22.60	51.07	35.78

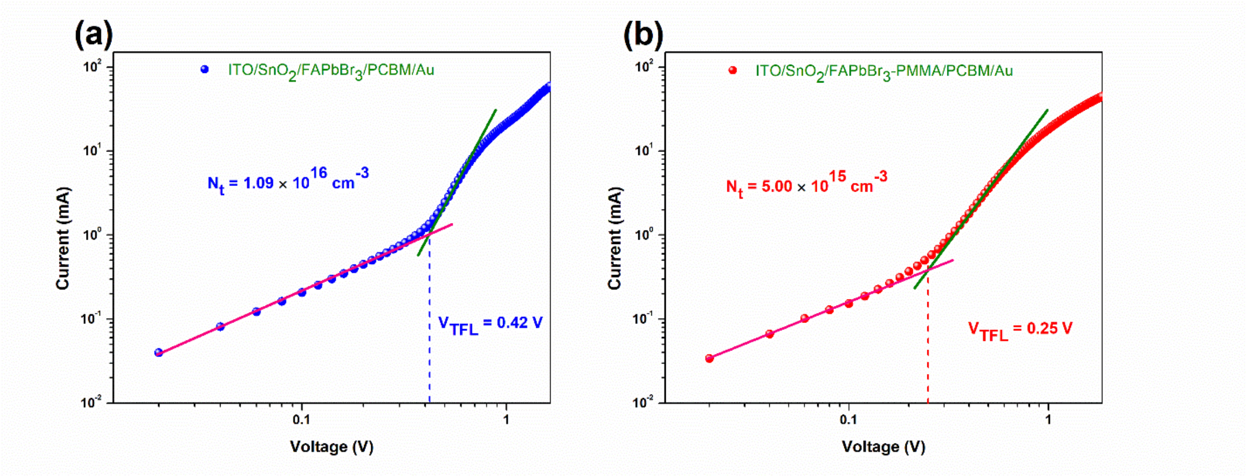

Figure S3: Space-charge-limited current (SCLC) measurements of electron-only devices fabricated with (a) untreated and (b) PMMA-treated $FAPbBr_3$ films, showing trap-filled limit voltages (V_{TFL}) and calculated trap densities (N_t).

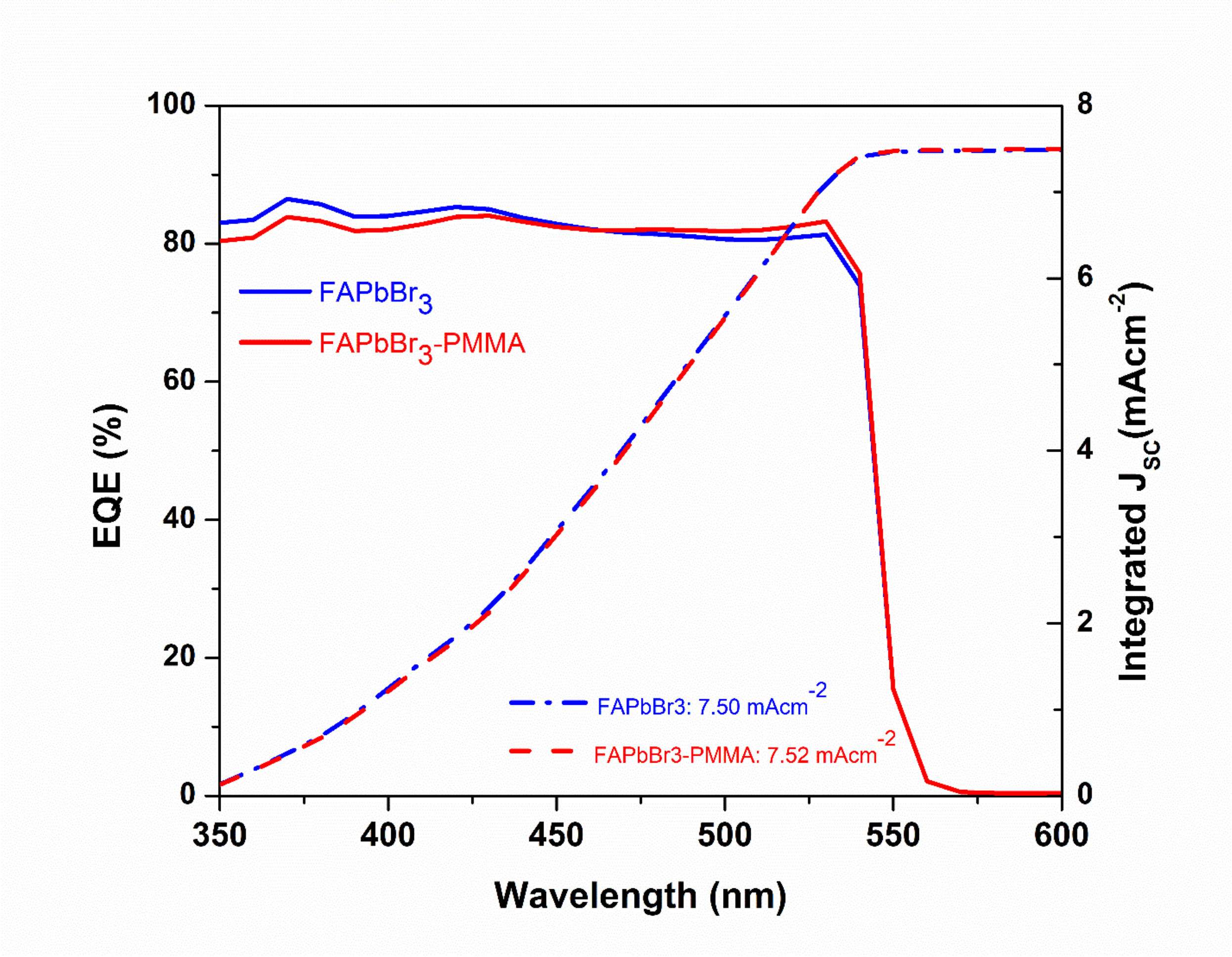

Figure S4: The external quantum efficiency (EQE) spectra and integrated J_{SC} for the best performing the $FAPbBr_3$ perovskite solar cells prepared without and with PMMA anti-solvent treatment.

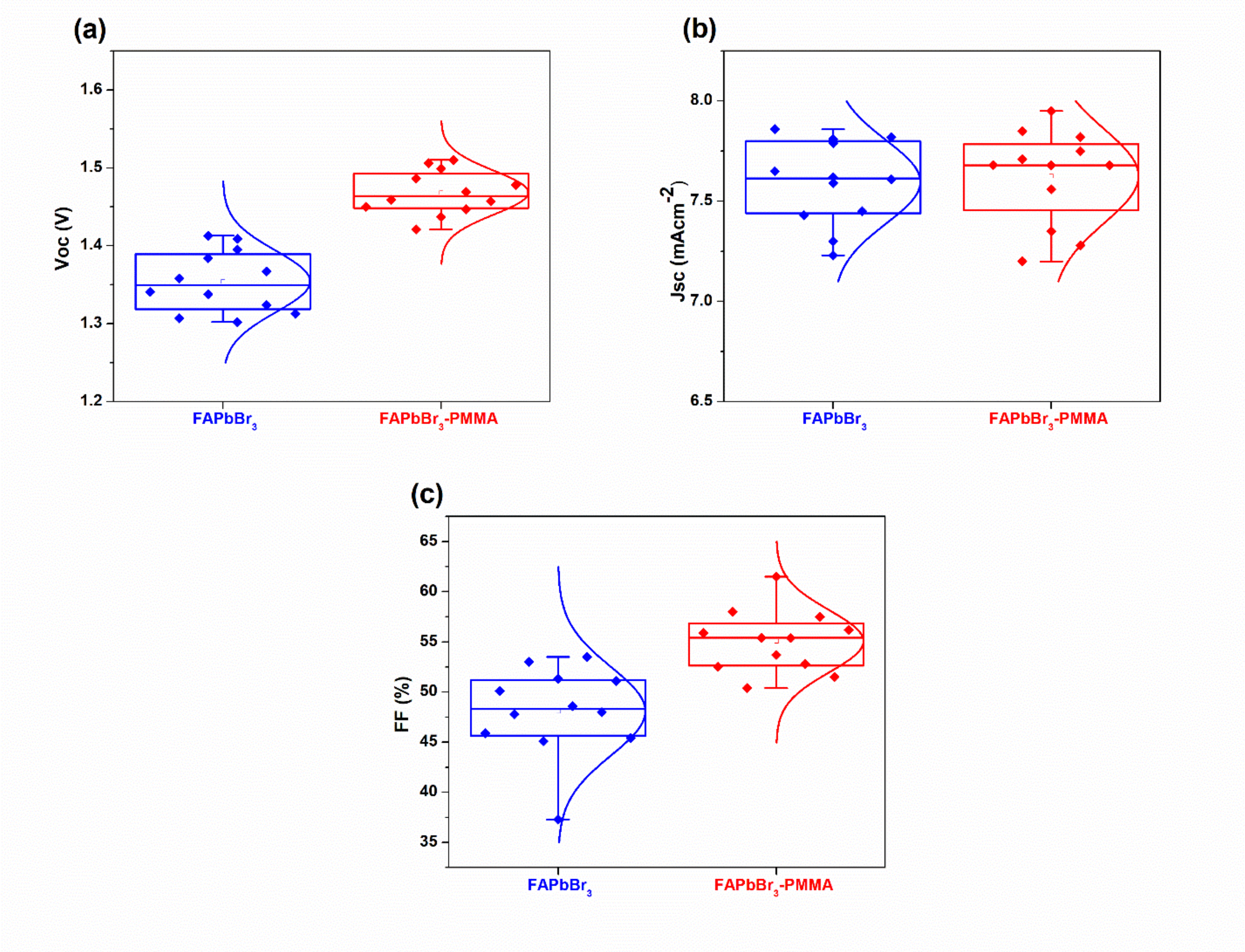

Figure S5: Statistical distribution box plots of (a)Voc, (b) Jsc and (c)FF for the $FAPbBr_3$ perovskite solar cells prepared without and with PMMA anti-solvent treatment.

Table S2: Summary of the average photovoltaic parameters of the $FAPbBr_3$ perovskite solar cells prepared without and with PMMA anti-solvent treatment.

Name	Voc (V)	Jsc(mA/cm^2)	FF(%)	PCE(%)
$FAPbBr_3$	1.354 ± 0.040	7.60 ± 0.21	48.1 ± 4.4	4.95 ± 0.55
FAPbBr3-PMMA	1.468 ± 0.028	7.63 ± 0.23	55.1 ± 3.1	6.16 ± 0.31

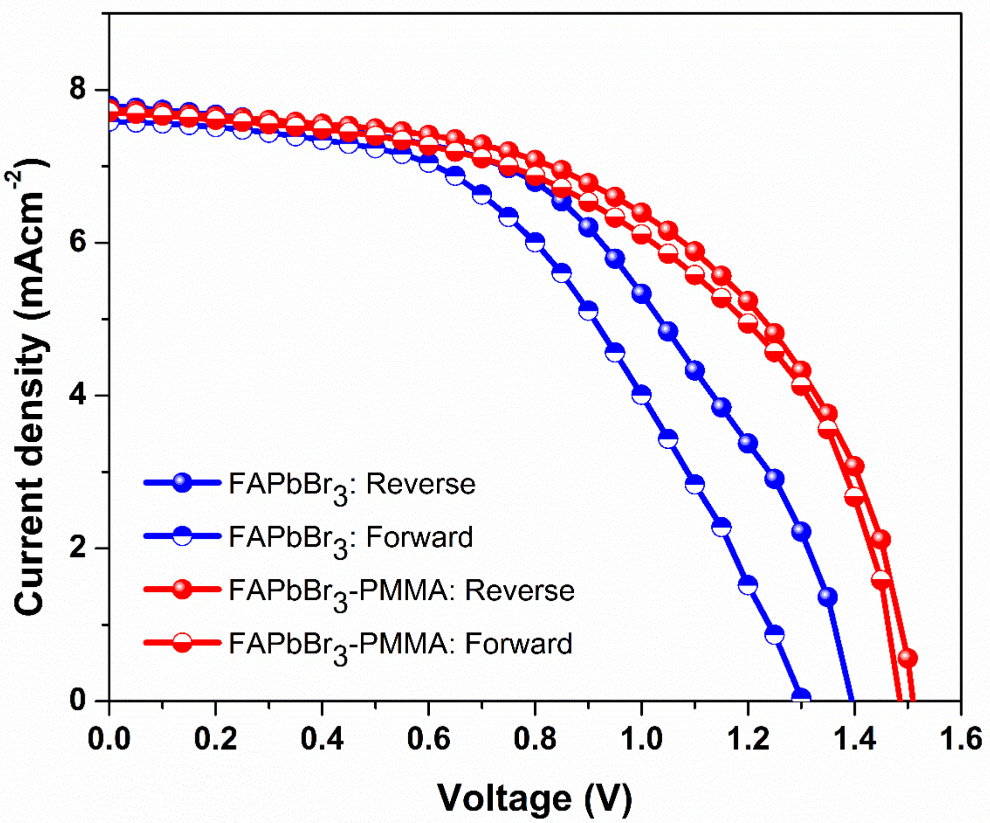

Figure S6: Current density-voltage (J-V) curves under reverse and forward scans the $FAPbBr_3$ perovskite solar cells prepared without and with PMMA anti-solvent treatment.

Table S3: Summary of the photovoltaic parameters of reverse and forward scans for the best performing $FAPbBr_3$ perovskite solar cells prepared without and with PMMA anti-solvent treatment.

Name	Voc (V)	Jsc (mA/cm^2)	FF (%)	PCE(%)	H-Index (%)
$FAPbBr_3$:Reverse	1.395	7.79	51.3	5.57	13.8 %
$FAPbBr_3$:Forward	1.302	7.59	48.6	4.80	
$FAPbBr_3$-PMMA: Reverse	1.510	7.75	55.4	6.48	5.1 %
$FAPbBr_3$-PMMA: Forward	1.486	7.71	53.7	6.15	

Table S4: Summary of the photovoltaic parameters of initial and after 4 weeks stored under ambient conditions for the $FAPbBr_3$ perovskite solar cells prepared without and with PMMA anti-solvent treatment.

Name	Voc (V)	Jsc (mA/cm^2)	FF (%)	PCE(%)	PCE-Retention(%)
$FAPbBr_3$:Initial	1.395	7.79	51.3	5.57	77.9 %
$FAPbBr_3$: After 4 weeks	1.311	7.01	47.2	4.34	
$FAPbBr_3$-PMMA: Initial	1.510	7.75	55.4	6.48	91.2 %
$FAPbBr_3$-PMMA: After 4 weeks	1.463	7.43	54.4	5.91	

Table S5: Comparison of key performance metrics for high-performing $FAPbBr_3$ perovskite solar cells reported in the literature.

**Device structure**	**Voc(V)**	**Jsc (mA/cm^2)**	**FF (%)**	**PCE (%)**	**Method**	**Ref**
FTO/TiO_2/$FAPbBr_3$/ Spiro-OMeTAD/Au	1.35	6.6	73	6.5	Two- step	[1]
FTO/ compact TiO_2/Li treated mesoporous TiO_2 / $FAPbBr_3$/spiro-OMeTAD/ Au	1.53	7.3	71	8.2	Two -step	[2]
FTO/d-TiO_2/$FAPbBr_3$/spiro-OMeTAD/Au	1.32	6.3	69	5.7	Single step	[3]
FTO/Compact Tio_2/meso TiO_2/$FAPbBr_3$/ HTL (Fluorene−Dithiophene) /Au	1.5	6.9	69	7.1	Two- step	[4]
FTO/PEDOT: PSS/$FAPbBr_3$(HBr)/PCBM/Zno	1.35	10.3	59	8.3	Single step	[5]
FTO/SnO_2/$FAPbBr_3$ (Urea)/ spiro-OMeTAD /Au	1.56	8.39	73	10.61	Two -step	[6]
FTO/TiO2/$Cs_{0.08}FA_{0.92}PbBr_3$/PTTA/Au	1.515	6.6	83.2	8.32	Two- step	[7]
FTO/SnO_2/$FAPbBr_3$/ PEABr/SpiroOMeTAD/Au	1.39	8.8	77.6	9.4	Two- steps	[8]
FTO/NiO/$FAPbBr_3$/Mg-ZnO/PCBM/BCP/Ag	1.44	8.92	71	9.06	Single step	[9]
FTO/TiO_2/Li-m-TiO_2/$FAPbBr_3$ /PMMA/spiro/Au	1.53	6.96	74	7.8	Two- steps	[10]
ITO/P3CT/FAPbBr3/TET/PCBM/C60/BCP/Cu	1.482	8.50	66.52	8.33	Two step	[11]
ITO/MoOx/P3CT/$FAPbBr_3$ (CsAc)/BCP/ /(AZO/C_{60}/ BCP)/Au	1.404	8.00	62.60	7.03	Two- step	[12]
ITO/SnO2/$FAPbBr_3$ (GABr)/Spiro-OMeTAD/Au	1.639	7.71	71	8.92	Single step	[13]
FTO/PEDOT:PSS/$FAPbBr_3$(MACl)/Spiro-OMeTAD/Au	1.38	8.6	78.04	9.26	Two -step	[14]
FTO/SnO_2/$FAPbBr_3$ (MACl)/carbon	1.571	8.35	82.8	10.86	Single step	[15]
ITO/NiOx/2PACz)/ FAPbBr3/C60/BCP/Ag	1.45	8.62	69.72	8.71	Single step	[16]
ITO/NiOx/PTTA / FAPbBr3/C60/BCP/Ag	1.43	8.63	61.25	7.52	Single step	[16]
FTO/ NiOx/2PACz/ perovskite/ Cl-TPPO/C_{60}/BCP/Ag	1.41	8.92	77.3	9.73	Single step	[17]
FTO/ NiO_x/1-2PACz/ perovskite/ Cl-TPPO/C60/BCP/Ag	1.51	9.16	80.6	11.4	Single step	[17]
FTO/TiO_2/SnO_2/$FAPbBr_3$ (BMIMBF4)/ ISO-ENO/PTTA/ITO	1.64	6.69	70.03	7.83	Single step	[18]
ITO/SnO_2/$FAPbBr_3$ (PMMA)/ Spiro-OMeTAD/Au	1.510	7.75	55.4	6.48	Single step	This work

References

[1] F. C. Hanusch *et al.*, "Efficient Planar Heterojunction Perovskite Solar Cells Based on Formamidinium Lead Bromide," *The Journal of Physical Chemistry Letters,* vol. 5, no. 16, pp. 2791-2795, 2014/08/21 2014.

[2] N. Arora *et al.*, "Intrinsic and Extrinsic Stability of Formamidinium Lead Bromide Perovskite Solar Cells Yielding High Photovoltage," *Nano letters,* vol. 16, 10/24 2016.

[3] S. Sarkar, J. Das, A. Subbiah, R. Singh, and N. Mahuli, "One-step Solution Processed FAPbBr3 Formation for Better Reproducible Planar Perovskite Solar Cell," *Energy Technology,* vol. 5, 07/31 2017.

[4] N. Arora *et al.*, "High Open-Circuit Voltage: Fabrication of Formamidinium Lead Bromide Perovskite Solar Cells Using Fluorene–Dithiophene Derivatives as Hole-Transporting Materials," *ACS Energy Letters,* vol. 1, 05/08 2016.

[5] A. S. Subbiah, S. Agarwal, N. Mahuli, P. Nair, M. van Hest, and S. K. Sarkar, "Stable p–i–n FAPbBr3 Devices with Improved Efficiency Using Sputtered ZnO as Electron Transport Layer," *Advanced Materials Interfaces,* vol. 4, no. 8, p. 1601143, 2017.

[6] Y. Zhang, Y. Liang, Y. Wang, F. Guo, L. Sun, and D. Xu, "Planar FAPbBr3 Solar Cells with the Power Conversion Efficiency above 10%," *ACS Energy Letters,* vol. 3, 07/02 2018.

[7] Y. Ko *et al.*, "Microtuning of the Wide-Bandgap Perovskite Lattice Plane for Efficient and Robust High-Voltage Planar Solar Cells Exceeding 1.5 V," *ACS Applied Energy Materials,* vol. 3, no. 3, pp. 2331-2341, 2020/03/23 2020.

[8] Y. Liu, B. J. Kim, H. Wu, G. Boschloo, and E. M. J. Johansson, "Efficient and Stable FAPbBr3 Perovskite Solar Cells via Interface Modification by a Low-Dimensional Perovskite Layer," *ACS Applied Energy Materials,* vol. 4, no. 9, pp. 9276-9282, 2021/09/27 2021.

[9] C. Hu, S. Benachigere Shivarudraiah, H. Sung, I. Williams, J. Halpert, and S. Yang, "Discovery of a New Intermediate Enables One-Step Deposition of High-Quality Perovskite Films via Solvent Engineering," *Solar RRL,* vol. 5, 02/01 2021.

[10] Y. Numata, N. Shibayama, and T. Miyasaka, "FAPbBr3 perovskite solar cells with VOC values over 1.5 V by controlled crystal growth using tetramethylenesulfoxide," *Journal of Materials Chemistry A,* 10.1039/D1TA08964A vol. 10, no. 2, pp. 672-681, 2022.

[11] S. Li, C. Deng, L. Tao, Z. Lu, W. Zhang, and W. Song, "Crystallization Control and Defect Passivation via a Cross-Linking Additive for High-Performance FAPbBr3 Perovskite Solar Cells," *The Journal of Physical Chemistry C,* vol. 125, no. 23, pp. 12551-12559, 2021/06/17 2021.

[12] L. Qian *et al.*, "High-performance FAPbBr3 perovskite solar cells using dual-function bathocuproine interlayer for surface passivation and energy level alignment," *Journal of Materials Science: Materials in Electronics,* vol. 33, no. 22, pp. 18028-18038, 2022/08/01 2022.

[13] H. Xu *et al.*, "Guanidinium-assisted crystallization modulation and reduction of open-circuit voltage deficit for efficient planar FAPbBr3 perovskite solar cells," *Chemical Engineering Journal,* vol. 437, p. 135181, 2022/06/01/ 2022.

[14] Y. Liu, B. Cai, H. Yang, G. Boschloo, and E. M. J. Johansson, "Solvent Engineering of Perovskite Crystallization for High Band Gap FAPbBr3 Perovskite Solar Cells Prepared in Ambient Condition," *ACS Applied Energy Materials,* vol. 6, no. 13, pp. 7102-7108, 2023/07/10 2023.

[15] W. Yue *et al.*, "Printable High-Efficiency and Stable FAPbBr3 Perovskite Solar Cells for Multifunctional Building-Integrated Photovoltaics," *Advanced Materials,* vol. 35, no. 36, p. 2301548, 2023.

[16] H. Zhu *et al.*, "δ-Phase Management of FAPbBr3 for Semitransparent Solar Cells," *Advanced Optical Materials,* vol. 11, no. 10, p. 2202827, 2023.

[17] H. Zhu *et al.*, "Improved Hole-Selective Contact Enables Highly Efficient and Stable FAPbBr(3) Perovskite Solar Cells and Semitransparent Modules," (in eng), *Adv Mater,* vol. 36, no. 33, p. e2406872, Aug 2024.
[18] B. Paci *et al.*, "Enhancing the Morpho-Structural Stability of FAPbBr3 Solar Cells via 2D Nanoscale Layer Passivation of the Perovskite Interface: An In-Situ XRD Study," *Nanomaterials,* vol. 15, no. 5, p. 327, 2025.
